# Calciphylaxis of the Postmenopausal Female Breast: An Uncommonly Encountered Mimic of Carcinoma

**DOI:** 10.1155/2017/4541620

**Published:** 2017-08-09

**Authors:** Aaron G. Novotny, Ashley B. Simpson, Melinda A. Kral, Benjamin C. Calhoun, Amy E. Cocco, Steven D. Billings, Susan K. Miller, Paulette L. Lebda, Charles D. Sturgis

**Affiliations:** ^1^Robert J. Tomsich Pathology and Laboratory Medicine Institute, Cleveland Clinic, Cleveland, OH 44195, USA; ^2^Surgery Institute, Breast Cancer Surgery, Cleveland Clinic, Cleveland, OH 44195, USA; ^3^Imaging Institute, Diagnostic Radiology, Breast Imaging, Cleveland Clinic, Cleveland, OH 44195, USA

## Abstract

Calciphylaxis is a serious medical condition that is typically associated with end-stage renal disease and presents as the sequelae of calcifications in arterioles with subsequent ischemia of affected tissues. Classically, calciphylaxis produces ulcerated and necrotic skin lesions. These may be biopsied to aid in confirmation of the diagnosis. Herein we report a case of a large necrotic breast lesion in the clinical setting of calciphylaxis, and we emphasize that a multidisciplinary approach to diagnosis and management is important to avoid unnecessary oncological resection.

## 1. Introduction

Calciphylaxis, or calcific uremic arteriolopathy, is a rare but morbid condition resulting from medial calcification of arterioles and leading to ischemia and resultant necrosis [1, 2]. In most instances, the pathogenesis of vascular calcifications in patients with calciphylaxis is related to chronic renal failure with subsequent dysregulation of calcium and phosphate homeostasis. As a result, diffuse calcification of the media and internal elastic lamina of small- to medium-sized arterioles occurs with intimal proliferation, arterial luminal diminution, and thrombosis causing tissue necrosis. While the classical clinical description of calciphylaxis includes cutaneous areas of erythematous tenderness with violaceous discoloration and hemorrhagic bullae over affected regions (commonly the lower legs), a few reports of calciphylaxis of the breast parenchyma do exist [3–11]. In some instances, calciphylaxis of the breast can lead to subacute or chronic changes of the skin overlying the breast including areas of ulceration, discoloration, and induration that may mimic inflammatory breast carcinoma [11, 12]. It is of paramount importance to exclude a locally advanced malignancy when a mass lesion with associated cutaneous changes is detected in the breast parenchyma of a postmenopausal female. We herein report a unique case in which information from the patient's history, physical examination, imaging, surgical consultative opinion, and core biopsy histology together allowed for a specific diagnosis of breast necrosis related to mammary calciphylaxis. Exclusion of a diagnosis of malignancy spared the patient an unnecessary oncologic resection.

## 2. Case Presentation

**Figure 1:**
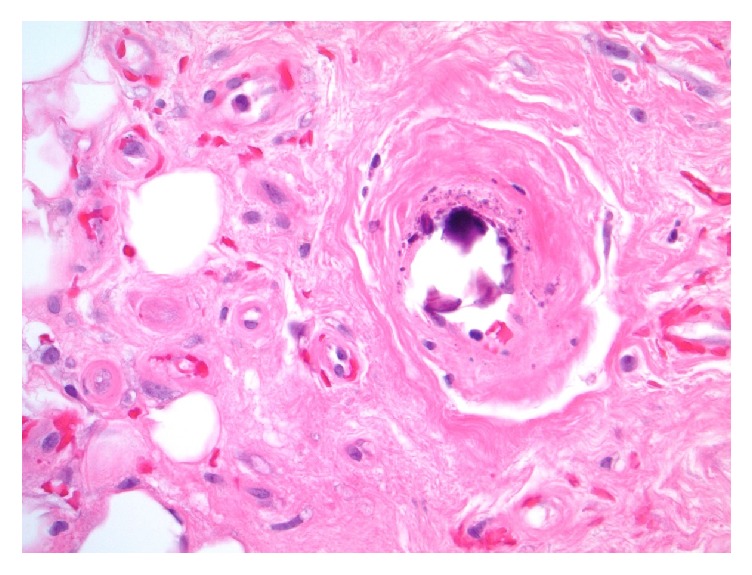
Photomicrograph from left leg skin punch biopsy showing calcification of dermal arteriole. Diagnostic of cutaneous calciphylaxis when combined with other histologic features and clinical setting (Hematoxylin & Eosin [H&E] stain, original magnification 400x).

The patient was a 54-year-old female who was admitted to hospital for management of hemodialysis related hypotension and treatment of a 6.5 cm, stage 4 decubitus ulceration of the skin and soft tissues around the coccyx. Her past medical history was remarkable for multiple comorbidities including stage 5 chronic kidney disease (end-stage renal disease), secondary hyperparathyroidism with associated renal osteodystrophy with lytic bone lesions, hypertension, chronic obstructive pulmonary disease, pulmonary hypertension, diabetes mellitus type II, paranoid schizophrenia, recurrent gastrointestinal bleeding, prior aortic valve endocarditis and regurgitation, prior complex endometrial hyperplasia without atypia, prior ischemic stroke, and prior deep venous thromboses. During her hospitalization, she was noted to have developed lower extremity skin changes with angulated retiform purpura of the upper lateral legs and worsening ulcerations with overlying scale crusts of the skin of the lower legs and thighs. Dermatology consultation was requested, and a clinical diagnosis of  “favor cutaneous calciphylaxis” was rendered. A skin punch biopsy from the left leg confirmed the diagnosis of calciphylaxis with histologic sections demonstrating atrophic epidermis with early changes of necrosis with overlying parakeratosis and serum crust. Numerous intravascular thrombi with associated extravasated erythrocytes and areas of ischemic necrosis were noted, and calcifications were confirmed both intrinsic to the walls of small blood vessels and within necrotic connective tissue (Figure 1).

**Figure 2:**
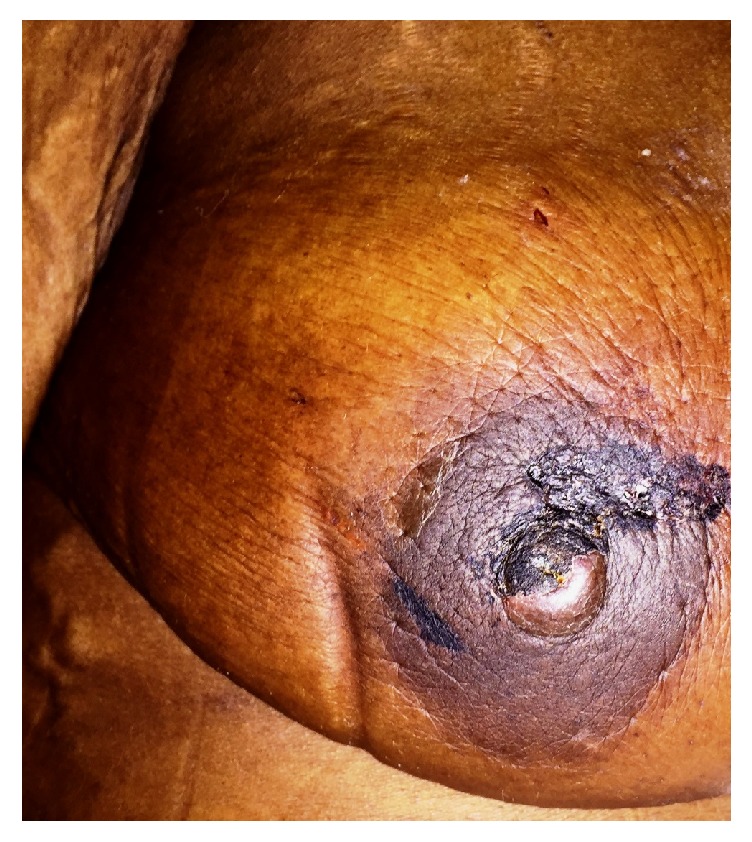
Photograph of the patient's right breast (taken prior to core biopsy) depicting a circumferential area of irregular hyperpigmentation surrounding the nipple-areolar complex with epidermal skin seepage of serous fluid and associated brown-black discoloration and scale crust covering portions of both the nipple and the areola.

**Figure 3:**
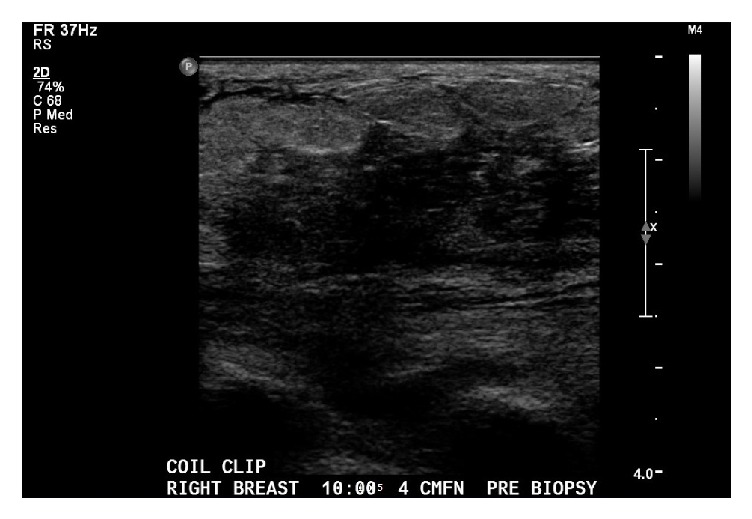
Ultrasound of ill-defined breast lesion showing hypoechoic mass with irregular borders. Ultrasound interpreted as BI-RADS 4, suspicious abnormality with differential diagnoses of phlegmon, malignancy, and calciphylaxis. Surgical consultation recommended.

**Figure 4:**
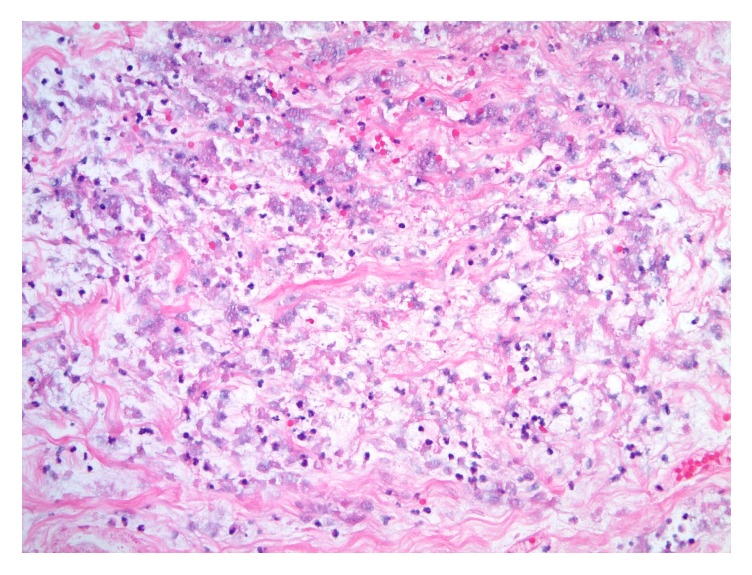
Photomicrograph of necrotic tissue in breast core biopsy with ghosted nuclei, extravasated erythrocytes, and a few scattered neutrophils (Hematoxylin and Eosin [H&E] stain, original magnification 200x).

A right breast mass with induration, erythema, mild pain, and a discolored area of skin encompassing the nipple-areola complex was also discovered on physical examination (Figure 2). No associated axillary or supraclavicular lymphadenopathy was identified at physical examination. A breast imaging consultation was requested. Color flow and real-time ultrasound examination of the right breast was performed, revealing a 6.8 × 4.8 × 2.1 cm lesion at 10 o'clock posterior depth, 4 cm from the nipple (Figure 3). This lesion was hypoechoic, showed no intrinsic vascularity, and was associated with the surrounding edema. The ultrasound findings correlated with the area of the patient's pain and cutaneous changes. The lesion was ultrasonographically labeled as a suspicious abnormality, BI-RADS 4, with a differential diagnosis of phlegmon (solid mass of inflamed connective tissue) versus malignancy versus calciphylaxis, and a surgical consultation was recommended. Surgical consultation resulted in a differential diagnosis of mammary calciphylaxis versus abscess versus malignancy. Because malignancy could not be entirely excluded, an ultrasound-guided core biopsy was performed. The breast core biopsy specimen consisted of two cylindrical portions of rubbery, tan tissue measuring 3.1 × 0.4 × 0.2 cm in aggregate. On histologic examination, a few millimeters of viable, nonneoplastic breast parenchyma with ducts, adipose tissue, and fibrous tissue could be identified at one tip of each core. The remainder of the core biopsy tissue (approximately 90% of the specimen) consisted of expanses of necrosis with no identifiable intrinsic epithelial structures, extravasated erythrocytes, and a few scattered neutrophils (Figure 4).

**Figure 5:**
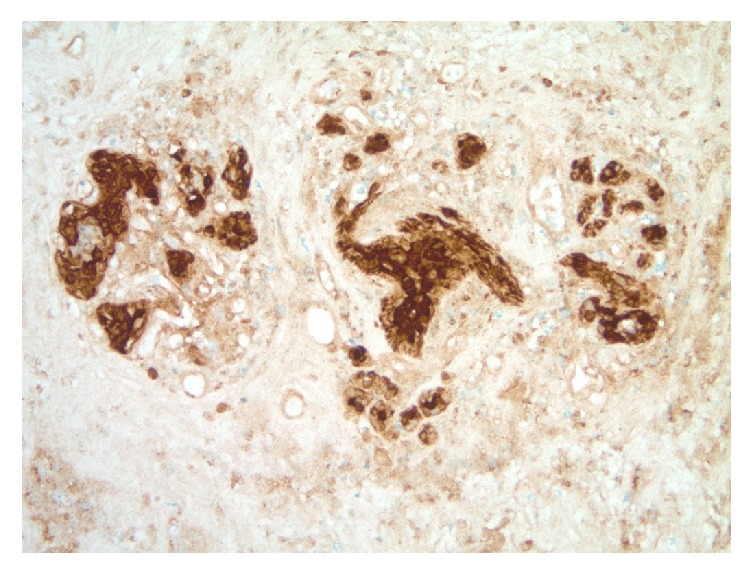
Photomicrograph of a ghosted terminal ductal lobular unit (TDLU) within breast core biopsy expanse of necrosis (CK7 immunohistochemical study, original magnification 200x).

**Figure 6:**
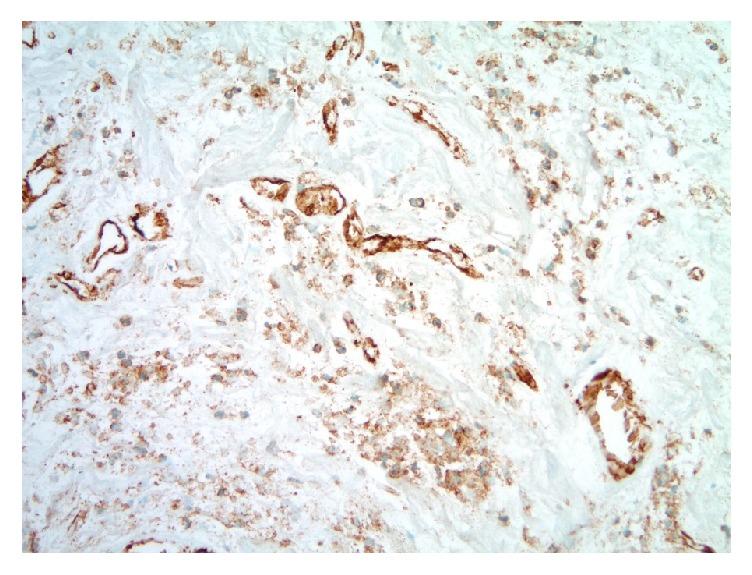
Photomicrograph of ghosted small blood vessel channels with breast core biopsy expanse of necrosis (CD31 immunohistochemical study, original magnification 200x).

**Figure 7:**
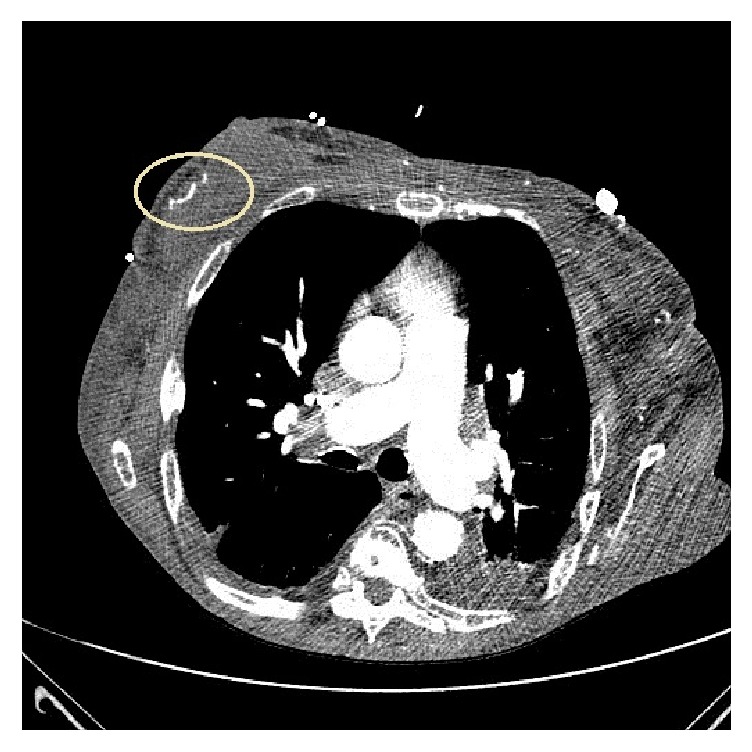
Axial CT of thorax (study performed for other reasons) demonstrating vascular calcifications intrinsic to the parenchyma of the patient's right breast and additionally supporting the clinical and histologic impressions of calciphylaxis.

No in situ epithelial proliferation or invasive carcinoma was histologically identified in the cores. With the differential diagnosis of ischemic necrosis versus tumor necrosis, ancillary immunohistochemical testing was performed. A cytokeratin 7 study highlighted a rare ghosted terminal ductal lobular unit (TDLU) within an expanse of necrosis and showed no evidence of sheet-like infiltrating neoplastic cells (Figure 5). Similarly, a CD31 study highlighted residual ghosted capillary-sized vascular channels (Figure 6). While frank calcific changes could not be identified in the breast cores by either hematoxylin and eosin stain or Von Kossa stain, the histologic pattern of extensive ischemic necrosis was felt compatible with the clinical setting of systemic calciphylaxis involving the substance of the right breast, and the immunohistochemical studies helped to further exclude an entirely necrotic malignancy. X-ray mammography of the breasts was not conducted at the time of the work-up of the necrotic breast mass, as the patient was debilitated/not able to stand. If mammography had been performed, a diffuse pattern of small vessel calcifications might have been of value in further supporting the diagnosis of calciphylaxis. Of note, a thoracic computed tomography study had been recently previously performed (for other reasons), and vascular calcifications were in retrospect noted within the substance of the right breast, additionally supporting the diagnosis of mammary calciphylaxis (Figure 7).

The combined imaging, surgical consultation, core biopsy, and pathological work-up in this patient allowed for exclusion of the differential diagnosis of malignancy, and the patient was spared an unnecessary oncologic resection. The patient's calciphylaxis was treated with sodium thiosulfate. The patient died seven months after evaluation of her breast disease. Her cause of death was multifactorial including multiorgan decline associated with multiple comorbidities including renal failure and systemic calciphylaxis. Exclusion of the differential diagnosis of a large invasive breast carcinoma allowed her clinical team to avoid an unnecessary major surgery and any associated potential adverse effects on length and quality of life.

## 3. Discussion

Calciphylaxis is an uncommon condition that is most often encountered in the clinical and pathological context of end-stage renal disease. It is classically characterized by skin ulcerations and necrosis and can be associated with significant pain. Calciphylaxis related wounds are prone to secondary infection and lead to death in up to 60% of patients within one year of diagnosis [13]. In addition to renal disease requiring dialysis, various other comorbidities have been described in the literature as being risk factors for calciphylaxis, including female sex, obesity, advanced age, hypertension, liver disease, diabetes mellitus, underlying malignancy, and hyperparathyroidism [13–15]. The incidence of calciphylaxis has increased in recent years with some estimates extending to as high as 5% of dialysis-dependent patients [13]. Whether the rise in incidence is due to improved recognition of the disease or to increased use of calcium based phosphate binders is uncertain. The exact prevalence of calciphylaxis is difficult to assess because of the difficulty in clinically distinguishing it from other overlapping cutaneous conditions that may be included in the differential diagnosis such as calcinosis cutis, warfarin-induced skin necrosis, primary vasculitides, ischemia from peripheral arterial occlusive atherosclerosis, cholesterol embolism syndrome, lipodermatosclerosis, and other forms of panniculitis [14].

Persons with advanced kidney disease who are treated with dialysis experience hyperphosphatemia, elevated calcium-phosphorous product, hypocalcemia, hyperparathyroidism, and often related vitamin D deficiency [15]. It is not entirely clear why most dialysis patients who receive “procalcification” therapies such as calcium salts and vitamin D do not develop calciphylaxis. While the role of dysregulated calcium-phosphorous metabolism as a risk factor for calciphylaxis cannot be overlooked, it appears not to be the sole or even most important relative risk. Demographic factors, comorbid conditions, and other medications may contribute to the risk of developing this condition. Research has elaborated the process by which calcium is deposited in vessel walls. It has been proposed that vascular lesional pathogenesis begins with the transformation of vascular smooth muscle cells into osteoblast-like phenotypes [16]. Induction of local hypercoagulability, involvement of regional inflammatory cytokines, lesional elevated bone morphogenetic protein-4 and osteopontin levels, and reactive oxygen species upregulating activators of nuclear transcription through loss of constituent inhibitors such as osteoprotegerin have also been linked to the development of calciphylaxis [17–19]. Of special relevance to practitioners of breast pathology are a few reported cases of mammary calciphylaxis following coronary artery bypass grafting and possible etiological roles of chemotherapy in promoting the development of calciphylaxis [20–22].

Because calciphylaxis is a systemic condition that may impact multiple organ systems, treatment often requires collaboration amongst multiple specialties including but not necessarily limited to nephrology, dermatology, wound care, nutrition, pain management, surgery, and psychiatry. Involvement of pathology early in the disease course is of paramount importance in establishing an accurate diagnosis upon which further therapeutic decision can be made. Treatments include correction of underlying calcium and phosphorous derangements, wound management, pain management, sodium thiosulfate use, hyperbaric oxygen, anticoagulation, nutritional optimization, cessation of corticosteroid use (unless critical for other conditions), and possibly kidney transplantation [13–15]. Owing to the pain and morbidity suffered by patients with progressive calciphylaxis, as well as less than ideal documented long term outcomes for these patients, early discussions regarding prognosis and end of life discussions may be warranted [13]. Our patient presented with a 6.8 cm painful lesion in the left breast. Collaborations between breast imaging, breast surgery, and breast pathology services resulted in an accurate diagnosis that resulted in a timely and accurate diagnosis of an uncommon condition in an unusual anatomic location. The pathologic diagnosis of mammary calciphylaxis spared the patient unnecessary additional invasive procedures and abated concerns about the possibility of a breast malignancy for both the clinical care team and the patient.
